# Vancomycin-Resistant Enterococci Outbreak, Germany, and Calculation of Outbreak Start

**DOI:** 10.3201/eid1402.070752

**Published:** 2008-02

**Authors:** Ulrich Sagel, Berit Schulte, Peter Heeg, Stefan Borgmann

**Affiliations:** * analyse BioLab GmbH, Linz, Austria; † University of Tübingen, Tübingen, Germany; ‡ Synlab Medical Care Centre Weiden, Weiden, Germany

**Keywords:** vancomycin resistance, cost analysis, disease outbreaks, Germany, dispatch

## Abstract

On the basis of a large outbreak of vancomycin-resistant *Enterococcus faecium* in a German university hospital, we estimated costs (≈1 million Euros) that could have been avoided by early detection of the imminent outbreak. For this purpose, we demonstrate an easy-to-use statistical method.

Recently, vancomycin-resistant *Enterococcus faecium* (VRE) has been detected with increasing frequency in Germany (*1*). Although some hospitals have reported only sporadic findings, others have been faced with extended outbreaks (*2*–*5*). Apart from threatening patient health, these pathogens have an unfavorable economic impact on resources for healthcare in general. Control of VRE has proven to be costly and time-consuming (*6*). We therefore examined to what extent early implementation of control measures could have prevented these undesirable consequences in an outbreak of VRE at a university hospital in southwestern Germany.

At this hospital ≈68,000 inpatients and 220,000 outpatients are treated each year. In 2003, 5 VRE-colonized patients were identified. In 2004, VRE were first detected in April. While at most 3 cases per month were observed through July, 8 colonized patients were found in August (Figure 1, panel** A**). The number of colonized patients remained relatively high in the following months. By the end of December, the cumulative number of patients with VRE was 48 (Figure 1, panel** B**). Although medical microbiologists were concerned about a possible outbreak as early as August, decision makers were reluctant to acknowledge a situation that needed action to be taken until January 2005. At that time, an infection control program was implemented. It included VRE screening in stool and anal/rectal swab samples from patients exhibiting an increased risk for VRE carriage (*2*). This program resulted in a sharp increase of detected cases to a total of 105 patients for February and March 2005 (Figure 1, panel** A**).

**Figure 1:**
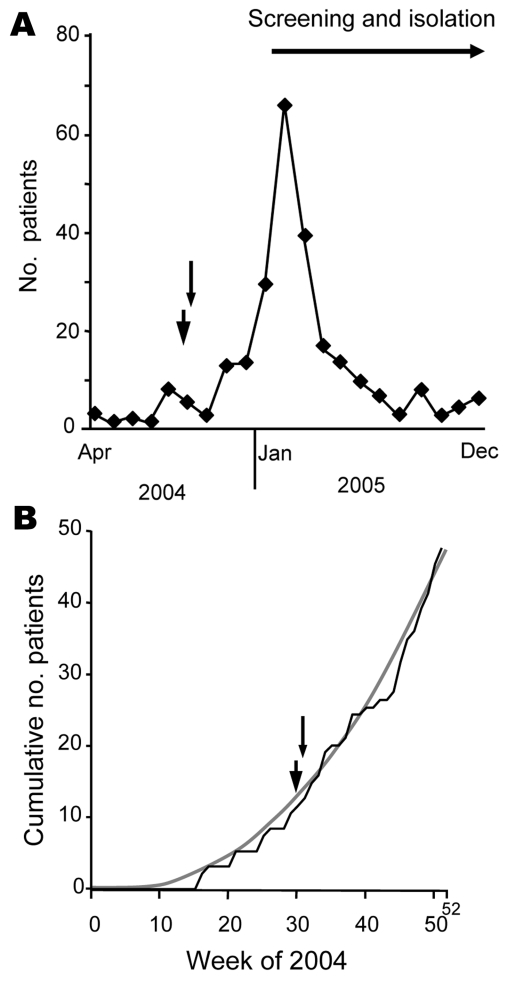
Course of vancomycin-resistant enterococci (VRE) outbreak at a German university hospital and time point (arrowhead, 30th calendar week; arrow, 31st calendar week) when outbreak alert could have been given. A) Number of VRE-carrying patients treated in a university hospital in 2004 and 2005. Given is the number of patients who were identified for the first time within a certain month (incident cases). In 2004 the first VRE patient was discovered in April 2005. B) Sum of VRE-exhibiting patients (cumulative number of patients [incident cases]) within distinct calendar weeks in 2004 (black line). Trend line (gray line) indicates exponential increase of numbers of incident cases (y = 0.002, χ^2^ – 0.3497 × 1.0299 [R^2^ = 0.9918]).

Retrospectively, we examined whether comprehensible proof of an imminent outbreak could have been given early enough to have convinced decision makers of the need for control measures. For early outbreak detection, we have chosen a simple approach that can be easily adopted by infection control nurses dealing with multiresistant pathogens usually occurring only sporadically (*7*). It is based on a Poisson distribution that describes the probability of rare, independent events in determined frames such as equal time periods (*8*). The course of a Poisson distribution is described by a complex formula containing several constants and 1 variable. This variable may be the number of VRE incidents that had happened in an earlier period (reference period). Knowing this, one can estimate the smallest number above a reference number to occur with a probability of p<0.05. Thus, for any small reference number, one can calculate a threshold number that cannot be explained by chance (respectively, a probability of p<0.05) and therefore suggests an imminent outbreak (Figure 2).

**Figure 2:**
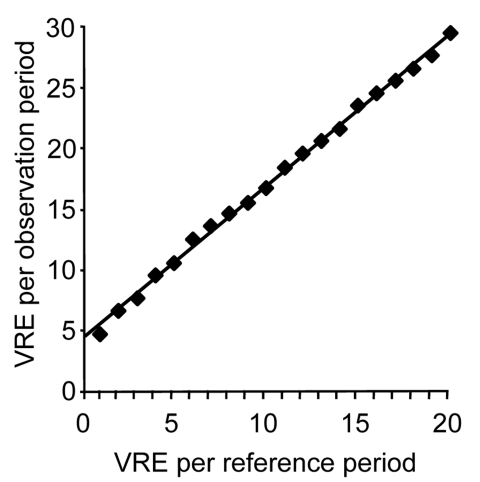
Alert threshold (p<0.05) derived from Poisson distribution; alert number for affected patients within an observation period depends on the number of patients found in a reference period. VRE, vancomycin-resistant enterococci.

Sporadic cases of peculiar multiresistant pathogens at a hospital within a calendar year would be such a rare event. In 2003, 5 cases of VRE were observed within 1 hospital; therefore, one would expect some number of cases close to 5 to appear in 2004 by chance. As shown in Figure 2, if one considers 5 cases as the reference number, the occurrence of 10 cases would justify an outbreak alert. If one accounts for some underreporting in the reference period and assumes 7 cases as reference, 12 cases would be the threshold. In this example, the 10th VRE case in 2004 appeared at the 30th calendar week, and the 12th case at the 31st calendar week.

Using this method, one must be aware of some limitations. Diagnostic awareness and procedures should not have changed within the compared periods. Furthermore, comparing calendar years, as shown in our example for ease, may lead to late alerts; we should have taken the first 30 (or 31) weeks of 2003 as a more appropriate reference period.

Two unusual aspects of our example deserve mention: First, the outbreak was caused by 2 strains (ST203, ST280) and not by 1 strain. While strain ST203 was found in various departments, ST280 VRE was isolated from patients in 2 adjacent intensive care units (*2*). Apparently, this did not jeopardize the usefulness of the outbreak alert method we used. Second, other hospitals in the region also had reports about clusters of VRE (*5*). Therefore, hygienic measures may also have been hampered by admission of some colonized patients from outside the hospital (*9*). Regardless of whether cases were caused by within-hospital transmission or by introduction from outside, a markedly increased number of cases requires attention and control measures to prevent further spread.

On the basis of our calculations, outbreak control measures could have been justified by a comprehensible statistical method in August 2004 (30th/31st calendar week). Instead, the cumulative numbers of cases increased exponentially and insidiously for 4 more months until the decision makers accepted the need for an infection control program (Figure 1, panel** B**). Fortunately, hygienic measures effectively reduced the numbers of incident cases (Figure 1, panel** A**). Nonetheless, the impact of dozens of cases was high, and it took strong efforts and several months to get the outbreak under control (i.e., only a few cases of VRE strain ST203 still prevalent in the hospital).

Complete calculation of costs compared with those that could have been prevented by an early outbreak alert is complicated and retrospectively hard to achieve. For a crude estimation of minimum of avoidable costs, we assume that taking measures as early as August 2004 would have led to controlling the outbreak by January 2005. Since control measures in 2005 have sharply reduced the number of incident cases to a low level (Figure 1, panel** A**), admission from outside appears to play a minor role.

Therefore, we examined VRE patients identified from February through March 2005 (n = 105). Conclusive data were obtained from 93 patients who were kept in isolation for a total of 2,631 days. In the hospital, only 2 rooms were available for individual patient isolation. Per isolation day, there would have been a loss of income of ≈615 Euros for unused beds due to rooms occupied for isolation measures. This would have added 1,618,065 Euros in a completely occupied hospital. However, in 2004 and also in 2005, the occupancy of the hospital was only ≈80%, which lowered the loss of income by 40%. With total occupancy, the loss of income would be reduced to 970,839 Euros. However, because total occupancy will not be achieved in practice, the real loss of income was some amount between 970,839 Euros and 1,618,065 Euros. It is sufficient to calculate the severe loss of income to illustrate that the economic effects of a large outbreak of VRE in a hospital can reach a loss of ≈1 million Euros. In a recent analysis of a VRE outbreak in Australia that resulted in the colonization of 64 patients, the costs were calculated at 2.7 million Australian dollars (*6*).

From our experience, we recommend making use of easy-to-use statistical models to detect and prevent imminent outbreaks as soon as possible. In this way, costs that may easily exceed 1 million Euros can be prevented.
